# Recent advances in managing hepatitis D

**DOI:** 10.12688/f1000research.11796.1

**Published:** 2017-08-30

**Authors:** Cihan Yurdaydin

**Affiliations:** 1 Department of Gastroenterology, University of Ankara Medical School, Ankara, Turkey; 2 Hepatology Institute, University of Ankara, Ankara, Turkey

**Keywords:** Hepatitis D Virus, pegylated interferon, hepatotrop

## Abstract

Hepatitis D virus (HDV) infection leads to the most severe form of chronic viral hepatitis and requires the attention of a liver specialist. In this review, I will recapitulate recent advances in the management of HDV, present background information on HDV infection as well as current chronic hepatitis D treatment, briefly examine the HDV life cycle and discuss new management strategies.

## Introduction

Hepatitis D virus (HDV) infection leads to the most severe form of chronic viral hepatitis and needs attention from a liver specialist in this context. Since its discovery in the 1970s by Mario Rizzetto and co-workers ^1^, it is well known that it cannot lead to liver disease on its own but needs the helper function of the hepatitis B virus (HBV). Only as a dual infection with HBV can HDV cause liver disease in humans. The helper function needed by HDV from HBV is hepatitis B surface antigen (HBsAg) synthesis. HDV needs HBsAg for virion assembly when the HDV nucleoprotein complex, formed in the nucleus and secreted into the cytoplasm, is covered by HBsAg to complete virion morphogenesis. The HBsAg envelop of the HDV virion is critical for cellular attachment and for the propagation of HDV to other hepatocytes ^2, 3^.

The aim of this review is to recapitulate recent advances in the management of HDV. For this, some background information for HDV infection in men, including current treatment of chronic hepatitis D (CHD), will be provided. New management strategies will be preceded by a short debrief of the HDV life cycle.

## HDV disease in humans

HDV leads to liver disease in humans either as co-infection with HBV or as superinfection of a chronic HBsAg carrier with HDV. The consequence of the former scenario is acute HDV infection. Acute HDV hepatitis may be associated with a biphasic clinical presentation, possibly related to sequential expression of the two viruses, observed both in early chimpanzee studies ^4^ and prospectively in intravenous drug addicts in men ^5^. Otherwise, acute HDV infection cannot be differentiated from other acute hepatotropic viral infections on clinical grounds. However, HDV infection may more often be associated with a severe course, and frequent occurrence of fulminant delta hepatitis has been reported both in the US and in Europe ^6, 7^. However, in recent years, fulminant delta hepatitis is less frequently encountered ^8^, which may be linked to the slower turnover of HDV in the community ^9^. The chronicity rate of HBV–HDV co-infection in adults appears to be no different than the chronicity development rate after acute hepatitis B mono-infection ^10^.

CHD is associated with the most severe form of hepatotropic-induced viral hepatitis ^11, 12^. However, the severity of the course of CHD may be dependent on host and viral factors, as CHD is an immune-mediated disease ^13^. Among viral factors, both HDV and HBV genotypes matter. Genotype II HDV seen in the Far East is associated with a milder form of chronic hepatitis compared to genotype I, which has a worldwide distribution ^14, 15^. Genotype III HDV is observed in the Amazon region of South America and is associated with a particular severe clinical presentation ^16^. Genotype IV, reported formerly as genotype IIb, has been seen both in the Far East (Japan and Taiwan) and in Africa ^17– 19^. Genotypes V to VIII are seen exclusively in Africa ^19, 20^. African CHD may be associated with a mild form of the disease and may respond better to interferon (IFN) treatment, as has been reported very recently ^21^.

In addition to HDV genotypes, HBV genotypes may also contribute to the clinical presentation of CHD. In a study from Brazil, HDV viral load was reported to be lower in genotype A compared to genotype D or F patients ^22^. In a study from Taiwan, HBV genotype C was linked to more severe disease compared to genotype B. Multivariate analysis identified age, HBV genotype C, and HDV genotype I as independent factors for poor outcomes ^14^.

Delta hepatitis has received the designation of orphan disease both in the US and in the European Union. This emphasizes that in both geographical regions, HDV infection is infrequently encountered. In such circumstances, it is not easy to follow expert advice to test every HBsAg carrier for HDV, which involves testing a cheap serological marker. However, forgetting to test may have dreadful consequences for patients. This is highlighted in a recent study from the US where, out of 1,191 chronic hepatitis B (CHB) patients, only 499 had been tested for anti HDV. Out of these 499 patients, 42 (8%) were anti HDV positive and among HDV co-infected patients 70% had cirrhosis ^23^. Therefore, in non-endemic areas, special attention to high-risk patients for acquiring HDV is needed. These are mainly intravenous drug users and immigrants from HDV-endemic countries, which may represent 55 to 95% of the total HDV population in Western European countries including Germany, Italy, Spain, Sweden, France, and England ^24– 31^. Endemic countries or regions for HDV include Mongolia, Pakistan, sub-Saharan Africa, Turkey, Romania, Albania, former Soviet republics, and the Amazon region in the northern part of South America ^32^.

## Current management of CHD

The management of CHD has not changed in over 30 years and consists of treatment with IFNs. The only modification in therapy is the switch from conventional to pegylated IFN (peg-IFN), which probably has not led to better viral response rates but merely has the comfort for patients of once-weekly dosing compared to the thrice-weekly schedule of conventional IFNs, thanks to the more suitable pharmacodynamic properties of peg-IFNs. Peg-IFN alpha 2a should be administered as subcutaneous injections at a dose of 180 μg once weekly and peg-IFN alpha 2b at a dose of 1.5 μg/kg, similar to the dosing schedule used before the era of directing acting antiviral in hepatitis C infection. The optimal duration of treatment is not known, but treatment for 1 year is the most-studied and -used treatment duration ^33^. Combination treatment of peg-IFN with lamivudine ^34, 35^, ribavirin ^36, 37^, adefovir ^38^, and tenofovir ^39^ was explored but was disappointing, as it did not lead to better viral response rates compared to IFN monotherapy. The only exception was peg-IFN–adefovir combination therapy, which was also not associated with higher viral response rates compared to peg-IFN monotherapy but interestingly the decline of quantitative HBsAg levels was more pronounced compared to peg-IFN monotherapy ^38^. It was hoped that a similar effect on HBsAg levels would also be seen in the HIDIT-2 study, which compared peg-IFN–tenofovir combination with peg-IFN alone, but no such effect was observed ^39^.

Treatment with nucleoside/nucleotide analogs (NAs), currently the treatment of choice for CHB, was ineffective in CHD where NAs were tested for a period of 6–12 months ^9, 33^. This can be attributed to the inefficacy of NAs on HBsAg levels, as production of HBsAg represents the only HBV function needed by HDV. In contrast, a median 6 years of tenofovir treatment was effective in HIV–HDV co-infected patients ^40^. The exact mechanism of efficacy of tenofovir in HIV–HDV co-infected patients is not clear but needs explanation. Longer duration of NA treatment may lead to decreased recycling of HBV DNA to the nucleus, thus decreasing the HBV covalently closed circular DNA (HBV ccc DNA) pool with consequent decreases in HBsAg levels ^41^. However, another explanation could be that the observed beneficial effect may have been a result of immune re-constitution secondary to effective antiretroviral therapy ^42^. Further studies would be helpful. One such study has to deal with prolonged NA treatment in CHD without HIV infection. Since prolonged NA treatment has been reported to lead to a decrease in quantitative HBsAg levels ^43^, this deserves to be explored.

## The HDV life cycle

HDV enters the hepatocyte after attachment of the HDV virion to the hepatocyte. Entry into the hepatocyte is similar to the entry of HBV, as both HBV and HDV cellular attachment is dependent on various common structures within the HBsAg where the pre-S1 domain of the L protein plays a key role ^44^. First, heparan sulfate proteoglycans on the hepatocyte membrane are approached by the HDV virion ^45, 46^ followed by attachment to the entry receptor sodium taurocholate co-transporting polypeptide (NTCP) ^47^. At this stage, the integrity of the amino acids in the pre-S1 region is crucial. Once inside the hepatocyte, HDV is uncoated and the ribonucleoprotein complex is directed to the nucleus where HDV replication occurs through a double rolling circle mechanism ^48^. Importantly, RNA polymerase needed during replication is a host enzyme, making it an unsuitable target for drug intervention, contrary to the hepatitis B or C viruses, which use their own polymerase product ^9, 12^. HDV RNA encodes for one protein, the small hepatitis D antigen (S-HDAg), which promotes HDV RNA replication. Another protein, large HDAg (L-HDAg), is synthesized as a result of an editing event, catalyzed by the host enzyme adenosine deaminase acting on double-stranded RNA (ADAR1) ^49^. L-HDAg inhibits HDV RNA replication but is crucial for virion assembly ^12^. The newly formed ribonucleoprotein complex is exported to the cytoplasm and is coated with the HBV envelop covering the L-, M-, and S-domains of HBsAg. The nucleoprotein complex HBsAg bond is possible through a cysteine-containing 4-amino-acid motif at the L-HDAg, which serves as a substrate for prenyltransferases. There are two prenyltransferases, farnesyltransferase and geranylgeranyltransferase. Of these two enzymes, farnesyltransferase is used by HDV. Through the latter enzyme, a 15-carbon farnesyl moiety is attached to the large delta antigen ^35^. The HBsAg cover of the ribonucleoprotein complex is a prerequisite for successful completion of virion morphogenesis in HDV ^50^. The new virion then exits the cell either via multivesicular bodies or by utilizing the subviral particle secretion pathway via the Golgi ^44^.

## Management of CHD with drugs to come

In the last 5 years, new drugs have been explored for the management of CHD. These drugs tailor various steps in the life cycle of HDV. However, as a general comment, it is clear that any management strategy leading to a functional or complete cure in HBV ^51^ would be beneficial in CHD as well. After all, the ideal surrogate marker for effective treatment is HBsAg clearance, and the reason we use quantitative HDV RNA testing as a surrogate in daily practice and not HBsAg clearance is that the latter is so rarely achieved. However, patients who have a lasting virologic response to treatment have a high chance of clearing HBsAg on long-term follow-up. In a study from our own institution, we were able to show that 37% of patients with virologic response to conventional or peg-IFN lost HBsAg during a median follow-up of 5 years ^52^.

New compounds currently explored with relevance to the management of CHD are depicted in Table 1. The main drivers among new compounds are hepatocyte entry inhibitors, farnesyltransferase inhibitors, and nucleic acid polymers (NAPs), which will be discussed below in more detail.

**Table 1.** New treatments in chronic hepatitis D.

Hepatocyte entry inhibitors
Farnesyltransferase inhibitors
Nucleic acid polymers
Small interfering RNAs
Immunological approaches: Toll-like receptor agonists, checkpoint inhibitors, hepatitis B virus vaccines

### Hepatocyte entry inhibitors

Myrcludex B, a myristoylated lipopeptide comprising 47 amino acids of the pre-S1-domain of the HBV L-surface protein, represents the first hepatocyte entry inhibitor. As pointed out earlier, myristoylation of glycine in the pre-S1-domain and 77 amino acids in the pre-S1-domain appears to be important for cellular attachment ^2, 53^. Myrcludex B was tested both in intravenous and in subcutaneous formulations ( Table 2) ^54^. A transient, asymptomatic, serum lipase elevation was observed in 5 out of 36 healthy volunteers. Conjugated bile acids increased without clinical consequences. It needs to be mentioned that bile acids have been implicated in cardiac arrhythmias ^55^. In addition, mild alterations of hematological parameters have been reported, but overall the administration of Myrcludex B was reported to be well tolerated ( Table 3) ^54, 56^. The subcutaneous route displayed 85% bioavailability ^54^. In the proof-of-concept study, 24 patients received either 48 weeks of peg-IFN monotherapy or 24 weeks of 2 mg/kg subcutaneous Myrcludex B daily for 6 months (after that, patients received 48 weeks of peg-IFN monotherapy) or 6 months of Myrcludex B plus peg-IFN followed by 6 months of peg-IFN. Myrcludex B led to a >1 log _10_ reduction in HDV RNA in 6 out of 7 patients at the end of 24 weeks’ treatment ^56^. One patient became HDV RNA negative on monotherapy with Myrcludex B. Combination of Myrcludex B with peg-IFN was more effective, and 5 out of 7 patients became HDV RNA negative. The delta decline in HDV RNA was 1.67, 2.17, and 2.59 log _10 _with Myrcludex B, peg-IFN monotherapies, and their combination, respectively. HDV RNA negativity with combination treatment in 5 out of 7 patients after 6 months of treatment is promising, and a synergistic effect for combination treatment has been suggested ^56^. The primary endpoint of the study, a decrease in HBsAg levels of 0.5 IU/mL at any time during the study ^56^, was not reached, however. Further studies with Myrcludex B with different dosing and treatment duration regimens are to be expected, and such a study is already ongoing.

**Table 2.** Characteristics of novel drug treatment for chronic hepatitis D.

Drug	Mode of action	Administration route	Phase of study
Myrcludex B	Interferes with hepatitis D virus entry into hepatocyte through sodium taurocholate co-transporting polypeptide inhibition	Subcutaneous, daily for 6 months, ± pegylated interferon (peg-IFN)	Ib, IIa
Lonafarnib	Farnesyltransferase inhibitor, inhibits virion assembly	Oral, 2 to 12 months, ± ritonavir ± peg-IFN	II
Rep-2139-Ca	Nucleic acid polymer, binds with high affinity to amphipathic proteins, which are required at various stages of the viral life cycle	Intravenous infusion, once weekly for 4–6 months ± peg-IFN	II

**Table 3.** Side effects of the hepatocyte entry inhibitor myrcludex B, the farnesyltransferase inhibitor lonafarnib, and nucleic acid polymers.

**Myrcludex B** • Lipase and amylase elevation in phase I but not in phase II study • Elevation of taurine- and glycine-conjugated bile acids without apparent clinical consequences • Thrombocytopenia, neutropenia, lymphopenia, and eosinophilia: generally mild, transient
**Lonafarnib** • Gastrointestinal toxicity (anorexia, nausea with or without vomiting, diarrhea, weight loss): dose dependent and in lower dose cohorts generally mild and well tolerated
**Nucleic acid polymers** • Hair loss, dysphagia, anorexia, dysgeusia, in hepatitis B study: related to heavy metal exposure at the trial site? • Administration route-related side effects: peripheral grade 1 hyperemia, fever, chills, and headache

Myrcludex B is a promising new drug for both hepatitis B and D. That new treatment is a much urgent need in CHD does not need further explanation. The following issues remain to be resolved: (i) optimal dose and duration for use in human CHD needs to be established; (ii) superiority of combination treatment of Myrcludex B with peg-IFN over peg-IFN monotherapy needs to be convincingly shown; (iv) safety appears to be of less concern, but the effect on bile acids has to be explored in a larger patient group; (v) efforts for replacing a daily subcutaneous formulation with an oral formulation should be seriously considered – this would enable long-term use.

### Farnesyltransferase inhibitors

Prenylation of large hepatitis delta antigen is required for HDV virion assembly. Farnesyltransferase inhibitors are specific for HDV ^57^, and preclinical studies revealed that they abolished HDV-like particle production *in vitro* ^58^ and *in vivo* ^59^. In the proof-of-concept study, the farnesyltransferase inhibitor lonafarnib (LNF) was tested for a duration of 28 days in 14 CHD patients. A dose-dependent reduction of HDV RNA was observed ^60^. LNF serum concentrations correlated with HDV RNA change. Some characteristics of LNF are provided in Table 2. This study was followed by ongoing studies from the University of Ankara Medical School, Hannover Medical School, and National Institutes of Health (NIH) in Turkey, Germany, and the US, respectively. Three different approaches have been explored in these sites. In Ankara, optimal dosing in combination with ritonavir (RTN) with or without peg-IFN was explored. In Hannover, a dose-escalating study for LNF in combination with RTN was conducted, and at the NIH, once-daily dosing of LNF with RTN was explored. In Ankara, first in the LOW-R1 (LNF with and without RTN) study, LNF was tested at the higher doses of 200 and 300 mg, bid, and in combination with peg-IFN or RTN ^61^. Boosting with RTN increased serum concentrations of LNF 4- to 5-fold. The combinations of LNF–RTN and LNF–peg-IFN led to serum HDV RNA declines of more than 3 logs _10_ IU/mL from baseline after 8 weeks of treatment ^61^. Studies on the optimal combination treatment regimen with LNF and RTN and triple therapy with LNF, RTN, and peg-IFN have been tested for different durations in the LOW-R2 study. In this latter study, the triple combination treatment consisting of 25 mg LNF bid with RTN 100 mg bid and peg-IFN appeared to combine best efficacy with tolerability ^62^. Three out of 5 patients were HDV RNA negative after 24 weeks of treatment. LNF at 25 or 50 mg bid dosing with RTN was as effective as all oral combination treatment. Five out of 14 (36%) patients had HDV RNA levels below the limit of quantification at 24 weeks of treatment. Combination of LNF at doses of 75 mg bid and higher as dual or triple therapy was not tolerated because of side effects and also did not lead to better antiviral efficacy ^62^. In the dose-escalation study in Hannover, patients started with a LNF/RTN dose of 50 mg/100 mg bid and the LNF dose was increased first to 75 mg and then to 100 mg bid at roughly 4-week intervals when patients tolerated the administered doses. Dose was not further increased after reaching LNF/RTN (100 mg/100 mg bid). Escalation to this ultimate dose was possible in 10 out of 15 patients. One patient became HDV RNA negative at the end of treatment and another had HDV RNA levels below the quantification level ^63^. Overall, it appears that off-treatment virologic response is lacking in the majority of patients in these studies. An LNF–RTN once-daily dosing combination regimen for 24 weeks was tested in a double-blind approach at the NIH ^64^. Three regimens were tested: LNF 50/75/100 mg qd with RTN 100 mg qd. After 24 weeks of treatment, 6 out of 21 patients had HDV RNA levels below 250 IU/mL. Interestingly, the low-dose group appeared to have better antiviral efficacy compared to higher doses. ALT normalized in all of these studies with LNF in 47 to 75% of patients. LNF is associated with dose-limiting side effects, mainly related to gastrointestinal toxicity. These side effects comprise anorexia, nausea, diarrhea, and weight loss ( Table 3). Finally, in the LOW-R2 study, 5 out of 27 patients receiving treatment with LNF-based regimens for 12 to 24 weeks experienced a post-treatment ALT flare, which led to HDV RNA suppression to levels below the level of quantification or to undetectable levels ^65^.

These studies performed in the US, Germany, and Turkey have provided substantial data regarding optimal dosing and duration and the optimal combination treatments as well as safety. A larger study using low-dose LNF in combination with RTN with or without peg-IFN now needs to be conducted. The above-mentioned studies have confirmed previous data in oncology patients that LNF is associated with dose-limiting gastrointestinal adverse events. Hence, based on the data mentioned above, when combined with RTN, an LNF dose exceeding 50 mg bid should probably not be considered. Strategies for efficacy off-treatment need to be further explored. Along this line, the superiority of LNF and RTN in combination with peg-IFN over peg-IFN monotherapy has to be explored in a very well-designed study. The notion that patients treated with LNF-RTN as all oral combination may have sustained viral efficacy also has to be explored. Finally, the possibility of inducing a post-treatment beneficial immunological flare has to be investigated in a patient cohort with compensated and less-advanced liver disease.

### Nucleic acid polymers

NAPs are sequence-independent phosphorothioated oligonucleotides. Their mechanism of action is not clear. They may affect virus secretion from hepatocytes, although earlier steps of the viral cycle may also be targeted by NAPs. They bind with high affinity to amphipathic protein structures. In preclinical studies in duck HBV infection, the NAP REP9-AC was effective both *in vitro* ^66^ and *in vivo* ^67^. The first phase I/II study in humans was performed in Bangladesh. Seven out of 8 HBeAg-positive CHB patients receiving REP-2055 were reported to have 2 to 7 log reductions in HBsAg and 3 of them to have cleared HBsAg. All 7 patients had developed anti-HBsAg titers. Serum HBV DNA decreased by 3 to 7 logs after 20 to 27 weeks of treatment ^68^. Three patients who met protocol defined early termination criteria (e.g. HBsAg loss or HBeAg loss) discontinued treatment at weeks 20 to 27. On long-term off-treatment, only 1 patient had viral and serologic rebound, whereas the other 2 patients continued to be HBV DNA and HBsAg negative. However, REP-2055 intravenous infusion was associated with fever, chills, and headache. REP 2139-Ca, reported to be more stable, was given once-weekly by intravenous infusion to 12 HBeAg-positive CHB patients. Nine responder patients (decrease in HBsAg and HBV DNA by 2 logs) received add-on peg-IFN or thymosin after week 20. During monotherapy, 9 patients had an HBsAg drop of 2 to 7 logs, with 3 becoming HBsAg negative. Similar declines were observed in serum HBV DNA levels. However, off-treatment viral rebound occurred in 7 out of 9 patients within 3 to 24 months ^68^. REP-2139-Mg and REP-2165-Mg, a 2139 derivative with reportedly improved tissue clearance, is being tested in combination with peg-IFN and tenofovir in 20 HBeAg-negative CHB patients pre-treated with a 6-month course of tenofovir alone. Initial data suggest similar declines in HBsAg levels ^69^. All of these studies have relevance to CHD, since HBsAg appears to be selectively targeted. In the only study in CHD with NAPs, REP-2139-Ca was given once weekly with add-on peg-IFN starting at week 15 for another 15 weeks in 12 CHD patients ^70^. Peg-IFN alone was then continued as monotherapy for another 33 weeks. Nine patients displayed declines of >3 logs in HBsAg levels. Post-treatment results were presented at the EASL meeting this year. Five out of 12 patients continued to be HBsAg negative 1 year off-treatment ^71^. Besides administration route-related side effects such as fever, chills, and peripheral hyperemia, other reported side effects include anorexia, hair loss, dysphagia, and dysgeusia, which were attributed by the investigators as being secondary to heavy metal exposure at the trial site ( Table 3). Large randomized controlled trials are now awaited where the efficacy and durability of treatment response can be assessed as monotherapy as well as combination treatment.

NAPs led to the most dramatic response rates in terms of HBsAg declines in both CHB and CHD patients after treatment durations not exceeding 6 months in most cases. HBsAg declines in CHD patients were associated with substantial HDV RNA declines. They reported on- and off-treatment HBsAg clearance with development of antibody to HBsAg in a subset of patients. However, their spectacular results also raised some concerns, not confined to some unusual adverse events such as dysphagia. Deficiencies of pilot studies may be inevitable and understandable. They need to be addressed in better-prepared and -conducted larger studies with clear stopping rules where monotherapy with the selected NAP is compared to NAP–peg-IFN combination treatment as an example. The route of administration of NAPs (intravenous infusion) also appears to be a hurdle for this treatment approach.

### Additional treatment approaches

An exciting concept is the use of small interfering RNAs (siRNAs). ARC-520, a siRNA designed to reduce all HBV transcripts via RNA interference, dose-dependently decreased HBsAg levels after one single injection in HBeAg-negative CHB patients in a phase IIa clinical trial ^72^. A multi-dose extension study (up to 12 doses once-monthly) of the same compound has been conducted. With multiple dosing, additional decline of HBsAg levels was observed, more so in HBeAg-positive than in HBeAg-negative patients ^73^. It was reported that ARC-520 was well tolerated, but the study was put on hold owing to toxicity problems related to the carrier molecule.

### Immune system-targeted approaches

Such approaches may have regained interest in CHB, but a breakthrough clinical success is still awaited. Therapeutic vaccination strategies have not been successful so far. New approaches include DNA vaccines ^74^, anti-HB immune complexes ^75^, and the use of immunologically active adjuvants such as beta-glucosylceramide ^76^. Toll-like receptors (TLRs) as inducers of type 1 IFN responses have always raised interest. Several preclinical studies confirmed their key role in the induction of the innate immune system ^77^. The oral TLR-7 agonist GS-9620 was well tolerated and led to the induction of peripheral ISG15 production in CHB patients ^78^. No effect on HBV DNA was observed, which may be attributable to short treatment duration.

Drugs targeting programmed death protein 1 (PD1) and its ligand (PD-L1) are receiving attention in both cancer immunotherapy and chronic viral hepatitis. Immune checkpoints are exploited by viral pathogens, as shown by T-cell exhaustion in these conditions. Blocking PD1 appears to be a rational goal to activate the HBV-specific T-cell response. In a phase Ib clinical study, the immune checkpoint inhibitor nivolumab was well tolerated and was associated with a significant decline in HBsAg levels after a single dose of nivolumab ^79^. Hepatotoxicity and off-target immune activation are concerns. The future of immune therapy may be using combination immune therapy such as a PD1 blocker with DNA immunization and NA treatment. Such an approach may deserve serious consideration ^80^.

siRNAs and various immunological treatment approaches represent scientifically sound treatment vehicles. They are based on a solid scientific background. We know, for example, from hepatitis B that off-treatment viral clearance necessitates tight immune control, hence an immune-based approach in controlling CHD makes sense. However, data showing viral efficacy are needed. The lack of success so far points to the complexity of the immunological process. Maybe better surrogate markers of immunological activity are needed beforehand. Similarly, siRNAs deserve attention, as with one formulation several siRNAs targeting different pre-genomic RNAs can be used. However, for both the immunological and the oligonucleotide approach, time is needed for further development of these approaches.

## Conclusion

CHD represents the only hepatotropic viral infection where new drugs have not entered clinical practice despite being the most severe form of chronic viral hepatitis. Current treatment with IFNs is unsatisfactory. Hence, new treatment strategies are urgently required. Hepatocyte entry inhibitors, prenylation inhibitors, and NAPs represent a glimmer of hope for patients suffering from CHD. It may be said that none of the new approaches are comparable in efficacy and safety to new drug treatments such as entecavir or tenofovir in hepatitis B and several very effective direct-acting antivirals in hepatitis C. However, their first-generation counterparts also had several deficiencies. From the practicing clinician’s and patient’s point of view, despite their shortcomings, these new treatment approaches for CHD have to be developed fast, since at the current stage treatment with IFNs is effective in only a small proportion of patients, and the situation is further complicated by the decreased motivation and willingness of physicians to consider prolonged courses of peg-IFN treatment and as such some patients who would have benefited from peg-IFN treatment lose their chance. It is difficult to say which of the approaches is going to be the best option for the treatment of CHD, but it is likely that one, two, or all of the drugs reported above will be used in combination with peg-IFN. As such, CHD will probably remain the one viral hepatitis infection where IFN treatment will continue to be a part of its management.

## References

[1] Rizzetto M Canese MG Aricò S: Immunofluorescence detection of new antigen-antibody system (delta/anti-delta) associated to hepatitis B virus in liver and in serum of HBsAg carriers. *Gut.* 1977;18(12):997–1003. 10.1136/gut.18.12.997 75123 PMC1411847

[2] Blanchet M Sureau C: Infectivity determinants of the hepatitis B virus pre-S domain are confined to the N-terminal 75 amino acid residues. *J Virol.* 2007;81(11):5841–9. 10.1128/JVI.00096-07 17376925 PMC1900317

[3] Schulze A Schieck A Ni Y: Fine mapping of pre-S sequence requirements for hepatitis B virus large envelope protein-mediated receptor interaction. *J Virol.* 2010;84(4):1989–2000. 10.1128/JVI.01902-09 20007265 PMC2812397

[4] Rizzetto M Canese MG Gerin JL: Transmission of the hepatitis B virus-associated delta antigen to chimpanzees. *J Infect Dis.* 1980;141(5):590–602. 10.1093/infdis/141.5.590 6989929

[5] Caredda F Rossi E d'Arminio Monforte A: Hepatitis B virus-associated coinfection and superinfection with delta agent: indistinguishable disease with different outcome. *J Infect Dis.* 1985;151(5):925–8. 10.1093/infdis/151.5.925 3989325

[6] Smedile A Farci P Verme G: Influence of delta infection on severity of hepatitis B. *Lancet.* 1982;2(8305):945–7. 10.1016/S0140-6736(82)90156-8 6127458

[7] Govindarajan S Chin KP Redeker AG: Fulminant B viral hepatitis: role of delta agent. *Gastroenterology.* 1984;86(6):1417–20. 6714570

[8] Buti M Homs M Rodriguez-Frias F: Clinical outcome of acute and chronic hepatitis delta over time: a long-term follow-up study. *J Viral Hepat.* 2011;18(6):434–42. 10.1111/j.1365-2893.2010.01324.x 20546496

[9] Yurdaydin C Idilman R: Therapy of Delta Hepatitis. *Cold Spring Harb Perspect Med.* 2015;5(10): pii: a021543. 10.1101/cshperspect.a021543 26253093 PMC4588130

[10] Caredda F Antinori S Re T: Course and prognosis of acute HDV hepatitis. *Prog Clin Biol Res.* 1987;234:267–76. 3628381

[11] Rizzetto M Verme G Recchia S: Chronic hepatitis in carriers of hepatitis B surface antigen, with intrahepatic expression of the delta antigen. An active and progressive disease unresponsive to immunosuppressive treatment. *Ann Intern Med.* 1983;98(4):437–41. 10.7326/0003-4819-98-4-437 6340574

[12] Yurdaydın C Idilman R Bozkaya H: Natural history and treatment of chronic delta hepatitis. *J Viral Hepat.* 2010;17(11):749–56. 10.1111/j.1365-2893.2010.01353.x 20723036

[13] Grabowski J Wedemeyer H: Hepatitis delta: immunopathogenesis and clinical challenges. *Dig Dis.* 2010;28(1):133–8. 10.1159/000282076 20460901

[14] Su CW Huang YH Huo TI: Genotypes and viremia of hepatitis B and D viruses are associated with outcomes of chronic hepatitis D patients. *Gastroenterology.* 2006;130(6):1625–35. 10.1053/j.gastro.2006.01.035 16697726

[15] Wu JC Choo KB Chen CM: Genotyping of hepatitis D virus by restriction-fragment length polymorphism and relation to outcome of hepatitis D. *Lancet.* 1995;346(8980):939–41. 10.1016/S0140-6736(95)91558-3 7564729

[16] Casey JL Niro GA Engle RE: Hepatitis B virus (HBV)/hepatitis D virus (HDV) coinfection in outbreaks of acute hepatitis in the Peruvian Amazon basin: the roles of HDV genotype III and HBV genotype F. *J Infect Dis.* 1996;174(5):920–6. 10.1093/infdis/174.5.920 8896491

[17] Ma SP Sakugawa H Makino Y: The complete genomic sequence of hepatitis delta virus genotype IIb prevalent in Okinawa, Japan. *J Gen Virol.* 2003;84(Pt 2):461–4. 10.1099/vir.0.18762-0 12560580

[18] Wang TC Chao M: Molecular cloning and expression of the hepatitis delta virus genotype IIb genome. *Biochem Biophys Res Commun.* 2003;303(1):357–63. 10.1016/S0006-291X(03)00338-3 12646211

[19] Radjef N Gordien E Ivaniushina V: Molecular phylogenetic analyses indicate a wide and ancient radiation of African hepatitis delta virus, suggesting a *Deltavirus* genus of at least seven major clades. *J Virol.* 2004;78(5):2537–44. 10.1128/JVI.78.5.2537-2544.2004 14963156 PMC369207

[20] Makuwa M Caron M Souquière S: Prevalence and genetic diversity of hepatitis B and delta viruses in pregnant women in Gabon: molecular evidence that hepatitis delta virus clade 8 originates from and is endemic in central Africa. *J Clin Microbiol.* 2008;46(2):754–6. 10.1128/JCM.02142-07 18077651 PMC2238085

[21] Spaan M Carey I Wang B: Outcome in chronic hepatitis delta: differences between African and non-African patients. *J Hepatol.* 2017;66(1 Supplement):S255–6. 10.1016/S0168-8278(17)30819-X

[22] Kiesslich D Crispim MA Santos C: Influence of hepatitis B virus (HBV) genotype on the clinical course of disease in patients coinfected with HBV and hepatitis delta virus. *J Infect Dis.* 2009;199(11):1608–11. 10.1086/598955 19388852

[23] Gish RG Yi DH Kane S: Coinfection with hepatitis B and D: epidemiology, prevalence and disease in patients in Northern California. *J Gastroenterol Hepatol.* 2013;28(9):1521–5. 10.1111/jgh.12217 23574043

[24] Navascués CA Rodríguez M Sotorrío NG: Epidemiology of hepatitis D virus infection: changes in the last 14 years. *Am J Gastroenterol.* 1995;90(11):1981–4. 7485005

[25] Ordieres C Navascués CA González-Diéguez ML: Prevalence and epidemiology of hepatitis D among patients with chronic hepatitis B virus infection: a report from Northern Spain. *Eur J Gastroenterol Hepatol.* 2017;29(3):277–83. 10.1097/MEG.0000000000000795 27902514

[26] Ji J Sundquist K Sundquist J: A population-based study of hepatitis D virus as potential risk factor for hepatocellular carcinoma. *J Natl Cancer Inst.* 2012;104(10):790–2. 10.1093/jnci/djs168 22423008

[27] Cross TJ Rizzi P Horner M: The increasing prevalence of hepatitis delta virus (HDV) infection in South London. *J Med Virol.* 2008;80(2):277–82. 10.1002/jmv.21078 18098143

[28] El Bouzidi K Elamin W Kranzer K: Hepatitis delta virus testing, epidemiology and management: a multicentre cross-sectional study of patients in London. *J Clin Virol.* 2015;66:33–7. 10.1016/j.jcv.2015.02.011 25866333

[29] Heidrich B Deterding K Tillmann HL: Virological and clinical characteristics of delta hepatitis in Central Europe. *J Viral Hepat.* 2009;16(12):883–94. 10.1111/j.1365-2893.2009.01144.x 19566789

[30] Gaeta GB Stroffolini T Chiaramonte M: Chronic hepatitis D: a vanishing Disease? An Italian multicenter study. *Hepatology.* 2000;32(4 Pt 1):824–7. 10.1053/jhep.2000.17711 11003629

[31] Piccolo P Lenci I Telesca C: Patterns of chronic hepatitis B in Central Italy: a cross-sectional study. *Eur J Public Health.* 2010;20(6):711–3. 10.1093/eurpub/ckp168 19884157

[32] Rizzetto M Ciancio A: Epidemiology of hepatitis D. *Semin Liver Dis.* 2012;32(3):211–9. 10.1055/s-0032-1323626 22932969

[33] Yurdaydin C: Treatment of chronic delta hepatitis. *Semin Liver Dis.* 2012;32(3):237–44. 10.1055/s-0032-1323629 22932972

[34] Canbakan B Senturk H Tabak F: Efficacy of interferon alpha-2b and lamivudine combination treatment in comparison to interferon alpha-2b alone in chronic delta hepatitis: a randomized trial. *J Gastroenterol Hepatol.* 2006;21(4):657–63. 10.1111/j.1440-1746.2006.04082.x 16677149

[35] Yurdaydin C Bozkaya H Onder FO: Treatment of chronic delta hepatitis with lamivudine *vs* lamivudine + interferon *vs* interferon. *J Viral Hepat.* 2008;15(4):314–21. 10.1111/j.1365-2893.2007.00936.x 18307594

[36] Gunsar F Akarca US Ersoz G: Two-year interferon therapy with or without ribavirin in chronic delta hepatitis. *Antivir Ther.* 2005;10(6):721–6. 16218171

[37] Niro GA Ciancio A Gaeta GB: Pegylated interferon alpha-2b as monotherapy or in combination with ribavirin in chronic hepatitis delta. *Hepatology.* 2006;44(3):713–20. 10.1002/hep.21296 16941685

[38] Wedemeyer H Yurdaydìn C Dalekos GN: Peginterferon plus adefovir versus either drug alone for hepatitis delta. *N Engl J Med.* 2011;364(4):322–31. 10.1056/NEJMoa0912696 21268724

[39] Wedemeyer H Yurdaydin C Ernst S: 96 weeks of pegylated-Interferon-alpha-2a plus tenofovir or placebo for the treatment of hepatitis delta: the HIDIT-2 study (abstr.) *Hepatology.* 2013;58:222–3. Reference Source

[40] Sheldon J Ramos B Toro C: Does treatment of hepatitis B virus (HBV) infection reduce hepatitis delta virus (HDV) replication in HIV-HBV-HDV-coinfected patients? *Antivir Ther.* 2008;13(1):97–102. 18389903

[41] Wursthorn K Lutgehetmann M Dandri M: Peginterferon alpha-2b plus adefovir induce strong cccDNA decline and HBsAg reduction in patients with chronic hepatitis B. *Hepatology.* 2006;44(3):675–84. 10.1002/hep.21282 16941693

[42] Arendt E Jaroszewicz J Rockstroh J: Improved immune status corresponds with long-term decline of quantitative serum hepatitis B surface antigen in HBV/HIV co-infected patients. *Viral Immunol.* 2012;25(6):442–7. 10.1089/vim.2012.0036 23131018

[43] Manesis EK Schina M Le Gal F: Quantitative analysis of hepatitis D virus RNA and hepatitis B surface antigen serum levels in chronic delta hepatitis improves treatment monitoring. *Antivir Ther.* 2007;12(3):381–8. 17591028

[44] Lempp FA Ni Y Urban S: Hepatitis delta virus: insights into a peculiar pathogen and novel treatment options. *Nat Rev Gastroenterol Hepatol.* 2016;13(10):580–9. 10.1038/nrgastro.2016.126 27534692

[45] Schulze A Gripon P Urban S: Hepatitis B virus infection initiates with a large surface protein-dependent binding to heparan sulfate proteoglycans. *Hepatology.* 2007;46(6):1759–68. 10.1002/hep.21896 18046710

[46] Sureau C Salisse J: A conformational heparan sulfate binding site essential to infectivity overlaps with the conserved hepatitis B virus a-determinant. *Hepatology.* 2013;57(3):985–94. 10.1002/hep.26125 23161433

[47] Yan H Zhong G Xu G: Sodium taurocholate cotransporting polypeptide is a functional receptor for human hepatitis B and D virus. *eLife.* 2012;1:e00049. 10.7554/eLife.00049 23150796 PMC3485615

[48] Taylor JM: Hepatitis delta virus and its replication. In: Fields BN, Knipe DM, Howley PM, eds. *Fundamental Virology*, 3 ^rd^edn. Phladelphia: Lippincott-Raven Publishers,1996;1235–44.

[49] Polson AG Bass BL Casey JL: RNA editing of hepatitis delta virus antigenome by dsRNA-adenosine deaminase. *Nature.* 1996;380(6573):454–6. 10.1038/380454a0 8602246

[50] Glenn JS Watson JA Havel CM: Identification of a prenylation site in delta virus large antigen. *Science.* 1992;256(5061):1331–3. 10.1126/science.1598578 1598578

[51] Zeisel MB Lucifora J Mason WS: Towards an HBV cure: state-of-the-art and unresolved questions--report of the ANRS workshop on HBV cure. *Gut.* 2015;64(8):1314–26. 10.1136/gutjnl-2014-308943 25670809

[52] Keskin O Tuzun A Kalkan C: Prolongation of interferon therapy increases maintained virologic response rates and improves the natural course of chronic delta hepatitis (abstr.). *Hepatology.* 2015;62: p1000A. Reference Source

[53] Gripon P Le Seyec J Rumin S: Myristylation of the hepatitis B virus large surface protein is essential for viral infectivity. *Virology.* 1995;213(2):292–9. 10.1006/viro.1995.0002 7491754

[54] Blank A Markert C Hohmann N: First-in-human application of the novel hepatitis B and hepatitis D virus entry inhibitor myrcludex B. *J Hepatol.* 2016;65(3):483–9. 10.1016/j.jhep.2016.04.013 27132172

[55] Rainer PP Primessnig U Harenkamp S: Bile acids induce arrhythmias in human atrial myocardium--implications for altered serum bile acid composition in patients with atrial fibrillation. *Heart.* 2013;99(22):1685–92. 10.1136/heartjnl-2013-304163 23894089

[56] Bogomolov P Alexandrov A Voronkova N: Treatment of chronic hepatitis D with the entry inhibitor myrcludex B: First results of a phase Ib/IIa study. *J Hepatol.* 2016;65(3):490–8. 10.1016/j.jhep.2016.04.016 27132170

[57] Glenn JS: Prenylation of HDAg and antiviral drug development. *Curr Top Microbiol Immunol.* 2006;307:133–49. 10.1007/3-540-29802-9_7 16903224

[58] Bordier BB Marion PL Ohashi K: A prenylation inhibitor prevents production of infectious hepatitis delta virus particles. *J Virol.* 2002;76(20):10465–72. 10.1128/JVI.76.20.10465-10472.2002 12239323 PMC136538

[59] Bordier BB Ohkanda J Liu P: *In vivo* antiviral efficacy of prenylation inhibitors against hepatitis delta virus. *J Clin Invest.* 2003;112(3):407–14. 10.1172/JCI17704 12897208 PMC166292

[60] Koh C Canini L Dahari H: Oral prenylation inhibition with lonafarnib in chronic hepatitis D infection: a proof-of-concept randomised, double-blind, placebo-controlled phase 2A trial. *Lancet Infect Dis.* 2015;15(10):1167–74. 10.1016/S1473-3099(15)00074-2 26189433 PMC4700535

[61] Yurdaydin C Idilman R Choong I: O118: Optimizing the prenylation inhibitor lonafarnib using ritonavir boosting in patients with chronic delta hepatitis. *J Hepatol.* 2015;62(Supplement 2):S252 10.1016/S0168-8278(15)30137-9

[62] Yurdaydin C Idilman R Keskin O: A phase 2 dose-optimization study of lonafarnib with ritonavir for the treatment of chronic delta hepatitis—end of treatment results from the LOWR HDV-2 study. *J Hepatol.* 2017;66(1 Supplement):S33–S34. 10.1016/S0168-8278(17)30327-6

[63] Wedemeyer H Port K Deterding K: A phase 2 dose-escalation study of lonafarnib plus ritonavir in patients with chronic hepatitis D: final results from the Lonafarnib with ritonavir in HDV-4 (LOWR HDV-4) study. *J Hepatol.* 2017;66(1 Supplement):S24 10.1016/S0168-8278(17)30310-0

[64] Koh C Surana P Han T: A phase 2 study exploring once daily dosing of ritonavir boosted lonafarnib for the treatment of chronic delta hepatitis – end of study results from the LOWR HDV-3 study. *J Hepatol.* 2017;66(1 Supplement):S101–2. 10.1016/S0168-8278(17)30464-6

[65] Yurdaydin C Idilman R Kalkan C: The prenylation inhibitor lonafarnib can induce post-treatment viral clearance in patients with chronic delta hepatitis resulting in alt normalization and regression of fibrosis. *J Hepatol.* 2017;66(1 Supplement):S259 10.1016/S0168-8278(17)30828-0

[66] Noordeen F Vaillant A Jilbert AR: Nucleic acid polymers inhibit duck hepatitis B virus infection *in vitro*. *Antimicrob Agents Chemother.* 2013;57(11):5291–8. 10.1128/AAC.01003-13 23939902 PMC3811297

[67] Noordeen F Vaillant A Jilbert AR: Nucleic acid polymers prevent the establishment of duck hepatitis B virus infection *in vivo*. *Antimicrob Agents Chemother.* 2013;57(11):5299–306. 10.1128/AAC.01005-13 23939904 PMC3811296

[68] Al-Mahtab M Bazinet M Vaillant A: Safety and Efficacy of Nucleic Acid Polymers in Monotherapy and Combined with Immunotherapy in Treatment-Naive Bangladeshi Patients with HBeAg+ Chronic Hepatitis B Infection. *PLoS One.* 2016;11(6):e0156667. 10.1371/journal.pone.0156667 27257978 PMC4892580

[69] Bazinet M Pantea V Placinta G: Update on safety and efficacy in the REP 401 protocol: REP 2139-Mg or REP 2165-Mg used in combination with tenofovir disoproxil fumarate and pegylated Interferon alpha-2a in treatment naïve caucasian patients with chronic HBeAg negative HBV infection. *J Hepatol.* 2017;66(1 Supplement):S256–7. 10.1016/S0168-8278(17)30821-8

[70] Bazinet M Pantea V Cebotarescu V: Update on the safety and efficacy of REP 2139 monotherapy and subsequent combination therapy with pegylated interferon alpha-2a in Caucasian patients with chronic HBV/HDV co-infection. *J Hepatol.* 2016;64(2 Supplement):S584–85. 10.1016/S0168-8278(16)01073-4

[71] Bazinet M Pantea V Cebotarescu V: One year follow-up and HBV RNA / HBcrAg analysis in the REP 301 Trial: REP 2139 and pegylated interferon alpha-2a in Caucasian patients with chronic HBV / HDV co-infection. *J Hepatol.* 2017;66(1):S96–7. 10.1016/S0168-8278(17)30452-X

[72] Yuen MF Chan HL Given B: Phase II, dose ranging study of ARC-520, a siRNA-based therapeutic, in patients with chronic hepatitis B virus infection (abstr.). *Hepatology.* 2014;60:LB–21.

[73] Yuen MF Liu K Chan HL: Prolonged RNA interference therapy with ARC-520 Injection in treatment naïve, HBeAg positive and negative patients with chronic HBV results in significant reductions of HBs antigen. *J Hepatol.* 2017;66(1):S27 10.1016/S0168-8278(17)30316-1

[74] Mancini-Bourgine M Fontaine H Scott-Algara D: Induction or expansion of T-cell responses by a hepatitis B DNA vaccine administered to chronic HBV carriers. *Hepatology.* 2004;40(4):874–82. 10.1002/hep.20408 15382173

[75] Fontaine H Kahi S Chazallon C: Anti-HBV DNA vaccination does not prevent relapse after discontinuation of analogues in the treatment of chronic hepatitis B: a randomised trial--ANRS HB02 VAC-ADN. *Gut.* 2015;64(1):139–47. 10.1136/gutjnl-2013-305707 24555998

[76] Mizrahi M Lalazar G Ben Ya'acov A: Beta-glycoglycosphingolipid-induced augmentation of the anti-HBV immune response is associated with altered CD8 and NKT lymphocyte distribution: a novel adjuvant for HBV vaccination. *Vaccine.* 2008;26(21):2589–95. 10.1016/j.vaccine.2008.03.026 18423947

[77] Zhang E Lu M: Toll-like receptor (TLR)-mediated innate immune responses in the control of hepatitis B virus (HBV) infection. *Med Microbiol Immunol.* 2015;204(1):11–20. 10.1007/s00430-014-0370-1 25550115 PMC4305100

[78] Gane EJ Lim YS Gordon SC: The oral toll-like receptor-7 agonist GS-9620 in patients with chronic hepatitis B virus infection. *J Hepatol.* 2015;63(2):320–8. 10.1016/j.jhep.2015.02.037 25733157

[79] Gane E Gaggar A Nguyen AH: A phase1 study evaluating anti-PD-1 treatment with or without GS-4774 in HBeAg negative chronic hepatitis B patients. *J Hepatol.* 2017;66(1):S26–7. 10.1016/S0168-8278(17)30315-X

[80] Liu J Zhang E Ma Z: Enhancing virus-specific immunity *in vivo* by combining therapeutic vaccination and PD-L1 blockade in chronic hepadnaviral infection. *PLoS Pathog.* 2014;10(1):e1003856. 10.1371/journal.ppat.1003856 24391505 PMC3879364

